# Inhibitory effect of HTLV‐1 infection on the production of B‐cell activating factors in established follicular dendritic cell‐like cells

**DOI:** 10.1002/iid3.432

**Published:** 2021-05-04

**Authors:** Ayuko Takatani, Hideki Nakamura, Kaori Furukawa, Yushiro Endo, Masataka Umeda, Toshimasa Shimizu, Shin‐ya Nishihata, Kyoko Kitaoka, Tatsufumi Nakamura, Atsushi Kawakami

**Affiliations:** ^1^ Division of Advanced Preventive Medical Sciences, Department of Immunology and Rheumatology Nagasaki University Graduate School of Biomedical Sciences Nagasaki Japan; ^2^ Department of Otolaryngology—Head and Neck Surgery Nagasaki University Hospital Nagasaki Japan; ^3^ Department of Social Work, Faculty of Human and Social Studies Nagasaki International University Sasebo Japan

**Keywords:** B‐cell activating factor, FDC, follicular dendritic cell, HTLV‐1, Sjögren's syndrome

## Abstract

**Introduction:**

The low frequency of ectopic germinal center in labial salivary glands of patients with human T‐cell leukemia virus type 1 (HTLV‐1) antibody‐positive Sjögren's syndrome (SS) suggests that HTLV‐1 has some effects on follicular dendritic cells (FDCs).

**Methods:**

We used flow cytometry, immunofluorescence, and enzyme‐linked immunosorbent assays (ELISAs) to investigate the direct effect of HTLV‐1 on B‐cell activating factors produced by established FDC like cells obtained from excised human tonsils. We then measured the serum B‐cell activating factor (BAFF) and C‐X‐C motif ligand (CXCL) 13 concentrations of the HTLV‐1‐seropositive SS patients and the HTLV‐1‐seronegative SS patients by ELISA.

**Results:**

Among the 31 isolated specimens, 22 showed morphological characteristics of FDCs. Day 2‐cultured specimens showed expressions of CD14, CD23, CD40, intracellular adhesion molecule‐1 (ICAM‐1), and vascular cell adhesion molecule‐1. After 2 weeks, 12 of these specimens expressed ICAM‐1, FDC, and fibroblast cell marker. Intracellular BAFF and CXCL13 were constitutively expressed regardless of stimulation. After direct coculture with the HTLV‐1‐infected T‐cell line HCT‐5 or MT‐2, the BAFF and CXCL13 expressions on the FDC‐like cells were decreased in accord with the increased number of HCT‐5 and MT‐2 cells with styliform change and without HTLV‐1 Gag protein expression. Interferons upregulated the concentration of BAFF (but not CXCL13) in the culture supernatant, which showed a declining trend under the presence of HCT‐5 or MT‐2. The serum concentrations of BAFF and CXCL13 in the HTLV‐1‐seropositive SS patients were lower than those of the HTLV‐1 seronegative SS patients.

**Conclusions:**

HTLV‐1 partially inhibited the BAFF and CXCL13 expressions of established FDC‐like cells.

## Graphical abstract

Expression of B‐cell activating factor (BAFF) was upregulated by addition of interferons. Although BAFF expression was inhibited by addition of human T‐cell leukemia virus type 1 infected cell line, HCT‐5 and MT‐2, the level was not inhibited by addition of control cell line, MOLT‐4.

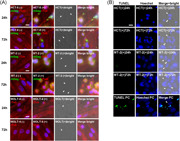

**Abbreviations**
ATL: adult T‐cell leukemia
BAFF: B‐cell activating factor
BLC: B‐lymphocyte chemoattractant
CXC: C‐X‐C motif ligand
eGC: ectopic germinal center
ELISA: enzyme‐linked immunosorbent assay
FCM: flow cytometry
FDC: follicular dendritic cell
FITC: fluorescein isothiocyanate
HAM: HTLV‐1‐associated myelopathy
HTLV‐1: human T‐cell leukemia virus type 1
HU: HTLV‐1‐associated uvitis
ICAM‐1: intracellular adhesion molecule‐1
ICOSL: inducible T‐cell co‐stimulator ligand
IF: immunofluorescence
IFN: interferon
LSG: labial salivary gland
LT: lymphotoxin
RA: rheumatoid arthritis
SS: Sjögren's syndrome
Tfh: follicular helper T cell
TNF: tumor necrosis factor
TRITC: tetramethyl rhodamine isothiocyanate
TUNEL: terminal deoxynucleotidyltransferase‐mediated dUTP nick end‐labeling
VCAM‐1: vascular cell adhesion molecule‐1

## 1 INTRODUCTION

In the production of autoantibodies, follicular dendritic cells (FDCs) are crucial in the selection of high‐affinity B cells.^1^, ^2^ FDCs in the outer lining of the ectopic germinal center (eGC) produce C‐X‐C motif ligand (CXCL) 13^3^ (also known as B‐lymphocyte chemoattractant [BLC]), which functions in the acceleration of B‐cell homing toward the eGC.^4^ CXCL13^−/−^ mice showed no accumulation of FDCs in the eGC,^5^ indicating that CXCL13 plays a crucial role in eGC formation. B‐cell activating factor (BAFF, or tumor necrosis factor [TNF] ligand superfamily member 13B) produced by FDCs selects high‐affinity B cells^5^, ^6^ with the potential to change to plasma cells that produce an autoantibody with the assistance of follicular helper T (Tfh) cells.^7^, ^8^, ^9^ CXCL13 and BAFF are thus considered the main factors in the maintenance of the B‐cell system in autoimmune function.

The retrovirus human T‐cell leukemia virus type 1 (HTLV‐1) causes adult T‐cell leukemia (ATL, which is a hematological neoplastic disorder), HTLV‐1‐associated myelopathy (HAM, which shows chronic progressive myelopathy), and HTLV‐1‐associated uvitis.^10^, ^11^, ^12^ Epidemiological studies revealed the prevalence of anti‐HTLV‐1 antibody in autoimmune diseases including rheumatoid arthritis and Sjögren's syndrome (SS), which is characterized by xerostomia, xerophthalmia, and the presence of autoantibodies including anti‐Ro/SS‐A and La/SS‐B antibodies.^13^, ^14^, ^15^ Our prior investigation of the epidemiological association between HTLV‐1 infection and SS revealed low prevalences of anti‐nuclear factor and anti‐Ro/SS‐A antibody in HTLV‐1‐seropositive patients with SS.^16^, ^17^ SS patients complicated with HAM showed a 30% prevalence of anti‐Ro/SS‐A antibody, whereas 70.4% of the HTLV‐1‐seronegative patients with SS had these autoantibodies.^17^

CXCL13 and its ligand CXCR5 are expressed in the eGC in the labial salivary glands (LSGs) of SS patients,^18^ indicating that the eGC can be a source of selected high‐affinity B cells in SS. However, we observed that anti‐HTLV‐1‐seropositive patients with SS had significantly low numbers of eGCs in their LSGs compared to anti‐HTLV‐1‐seronegative patients with SS, and the LSGs of the HAM‐SS patients showed no eGC formation, suggesting that HTLV‐1 might inhibit the formation of the eGC and related immune cells.^19^ Herein, we isolated FDC‐like cells from human tonsil tissues and characterized their phenotypic markers, and we examined the direct and indirect effects of HTLV‐1 infection on BAFFs (including CXCL13 and BAFF) in parallel with observations of these molecules on HTLV‐1‐infected cell lines.

## 2 MATERIALS AND METHODS

To establish FDC‐like cells, we used tonsil tissues from patients less than 10 years old. These tissues were surgically excised due to tonsillitis at our Department of Otolaryngology—Head and Neck Surgery. Each patient's parent/guardian provided informed consent. The use of human tissue was approved by the Ethics Committee (Human Studies) of Nagasaki University Hospital (No.: 16031412‐2).

We also used serum samples from 9 patients with HTLV‐1‐seropositive SS (1 male, 8 females; 63.0 ± 6.7 years), 30 patients with HTLV‐1‐seronegative SS (3 males, 27 females; 56.6 ± 15.5 years, without accurate SS disease duration information), and 13 patients without SS (1 male, 12 females; 58.1 ± 13.6 years) who were age‐ and sex‐matched to the HTLV‐1‐seronegative SS patients and had no immunological background. The use of SS patients' sera was also approved (No.: 20021018). An opt‐out disclosure was employed for the use of preserved human samples.

The SS patients fulfilled the 2002 American‐European Consensus Group classification criteria^20^ or the 1999 revised Japanese Ministry of Health criteria for SS.^21^

### 2.1 Antibodies and reagents

Table S1 provides the primary antibody details. Mouse anti‐CD14, CD23, intercellular adhesion molecule‐1 (ICAM‐1), vascular cell adhesion molecule‐1 (VCAM‐1), and BAFF/BLyS/TNFSF13B monoclonal antibodies were from Novus Biologicals (Centennial, Co.). Mouse antifibroblast surface protein, amphotericin B solution, and Hoechst dye 33258 were from Sigma‐Aldrich. Mouse anti‐CD40 antibody, mouse anti‐CXCR5 antibody, goat anti‐CXCL13 polyclonal antibody, recombinant interferon gamma (IFN‐γ), TNF‐α, and lymphotoxin α1β2 were from R&D Systems. Recombinant IFNα was from PBL Assay Science. Fluorescein isothiocyanate (FITC)‐conjugated mouse anti‐CD14, CD21, CD23, ICAM‐1, and mouse immunoglobulin G 1 (IgG1) were from Biolegend. Rabbit antialpha‐tubulin antibody was from Proteintech Group. FITC‐conjugated anti‐BAFF antibody was from ENZO Biochem. Alexa Fluor488‐conjugated anti‐CXCL13 antibody and FITC‐conjugated FDC antibody (CNA42) were from Novus Biologicals. Alexa Fluor488‐conjugated mouse IgG1 was from R&D Systems. FITC‐conjugated rat IgG2a was from eBioscience. Mouse IgG1, IgG2b, IgM, normal goat serum, normal rabbit serum, and mouse CD3 and CD4 monoclonal antibodies were from Dakocytomation. Donkey anti‐mouse IgG conjugated with FITC antibody (H + L), donkey anti‐rabbit/goat IgG conjugated with tetramethyl rhodamine isothiocyanate (TRITC) antibody (H + L), and donkey anti‐mouse IgM μ‐chain conjugated with FITC antibody were from Jackson ImmunoResearch Laboratories. Enzyme‐linked immunosorbent assay (ELISA) kits including BAFF/BLyS/TNFSF13B and human CXCL13/BLC/BCA‐1 Quantikine ELISA kits were from R&D Systems. The apoptosis inducer set including 10 mM actinomycin D, 5.7 mM camptothecin, 100 mM cycloheximide, 10 mM dexamethasone, and 100 mM etoposide was from BioVision Research Products. The MEBSTAIN Apoptosis TUNEL Kit Direct was from Medical & Biological Laboratories.

### 2.2 Cell lines

We used the cell line HCT‐5, which is a CD4^+^ T‐cell line from the cerebrospinal fluid of a HAM patient^21^, ^22^ maintained in RPMI 1640 culture medium containing interleukin (IL)‐2, 20% fetal bovine serum (FBS), and penicillin/streptomycin. The cell line MT‐2, which is derived from a patient with ATL, was maintained in RPMI 1640 culture medium containing 20% FBS. HTLV‐1‐seronegative MOLT‐4 cells that were derived from human acute lymphoblastic leukemia and maintained in RPMI 1640 culture medium containing 10% FBS were used as a control for the HCT‐5 and MT‐2 cells. The cells were continuously cultivated by replacing the culture medium three times per week.

### 2.3 Isolation and cell culture of FDC‐like cells

To establish FDC‐like cells, we used Muñoz‐Fernández et al.'s^24^ method with modification. Excised tonsil tissues were vigorously washed with phosphate‐buffered saline (PBS) and minced with scalpels. The cell suspension was then incubated in Collagenase V with DNase for 60 min, followed by filtering through gauze and centrifuging at 425*g* for 10 min. The cell pellet suspended in PBS was subjected to separation on Ficoll‐Paque media.

The interface was transferred to cell cultures with 10% FBS/RPMI with 100 UI/ml penicillin, 100 IU/ml streptomycin, and 0.25 μg/ml amphotericin. After we confirmed the attachment of adherent cells, nonadherent cells were discarded and the culture medium was replaced in 100‐mm^2^ dishes three times per week.

### 2.4 Flow cytometry (FCM) for FDC‐like cells

After the FDC‐like cells were treated with Gibco® TrypLE™ Express reagent (Thermo Fisher Scientific), we performed a FCM analysis to determine the expressions of the cell surface molecules and intracellular molecules. For fixation and permeabilization, a BD Cytofix/Cytoperm™ kit (BD Biosciences) was used.

For CXCL13 intracellular staining, FDC‐like cells were incubated for 12 h in Brefeldin A (BD Bioscience), which inhibits the protein transport to the Golgi complex. The primary antibodies for the FCM analysis were FITC‐conjugated anti‐CD14, anti‐CD21, anti‐CD23, ICAM‐1, and BAFF human monoclonal antibodies (Novus Biologicals). FITC‐conjugated mouse IgG1 was used as an isotype control. The experiments were performed with a FACS Canto II Flow Cytometer and FACS Diva software (BD Biosciences).

FDC‐like cells were treated with/without 1 μg/ml of IFN‐α and/or IFN‐γ or 10 ng/ml of TNF‐α and/or LTα1β2 for 24–72 h. After the TrypLE Express treatment, a subsequent Fc block (Human BD Fc Block™; BD Biosciences) was performed. FDC‐like cells were incubated with primary antibodies followed by FITC‐conjugated secondary antibodies when the primary antibodies were the nonconjugated type. Cell viability was measured by using the Zombie Aqua™ fixable viability kit (Biolegend). The data were analyzed using FlowJo™ ver. 10.7.1 software (FlowJo), and the degree of fluorescence was quantified as the geometric mean fluorescence intensity (GMFI).

### 2.5 Immunofluorescence (IF) analysis of FDC‐like cells

For the IF analysis of FDC‐like cells, we used Day‐2 FDC‐like cells from seven tonsillectomies. Cultured FDC‐like cells in a 100‐mm^2^ dish were trypsinized with Gibco 0.05% Trypsin‐EDTA (Thermo Fisher Scientific) and transferred to a 24‐well dish on 12‐mm^2^ cover slips. For Day‐2 specimens, FDC‐like cells were directly cultured on cover slips in 24‐well plates. The primary antibodies and isotype control were mouse anti‐CD14, CD23, CD40, ICAM‐1, VCAM‐1, and BAFF antibodies, and mouse IgG1 (working dilution 1:100).

After incubation with primary antibodies and isotype control followed by a wash with PBS, the FDC‐like cells were reacted with secondary antibodies including donkey anti‐mouse IgG conjugated with FITC antibody and Hoechst dye 33258 for 45 min at room temperature in the dark. The FDC‐like cells on 12‐mm^2^ cover slips were finally mounted in Vectashield mounting medium (Vector Laboratories). The stained images were captured by a fluorescence microscope (BZ‐X710; Keyence). A hybrid cell count system that was mounted on this microscope was used to quantify the mean fluorescence intensity (MFI).

### 2.6 Terminal deoxynucleotidyltransferase‐mediated dUTP nick end‐labeling (TUNEL) staining

For the identification of double‐stranded DNA breaks, we performed TUNEL staining for FDC‐like cells cocultured with HCT‐5 cells or MT‐2 cells. After fixation in 4% paraformaldehyde, FDC‐like cells were incubated in 50 μl of terminal deoxynucleotidyl transferase (TdT) solution including TdT buffer, FITC‐dUTP, and TdT at 37°C for 1 h using the MEBSTAIN Apoptosis kit Direct kit (MBL). After the cells were washed three times with distilled water, counterstaining was performed by immersing the cells in Hoechst 33258 solution for 20 min. The FITC signal of dUTP was captured by fluorescence microscopy using the BZ‐X710 microscope.

### 2.7 ELISAs for BAFF and CXCL13

The supernatant for the ELISAs was collected from 24‐well culture plates in which FDC‐like cells were directly cocultured with HCT‐5 cells, MT‐2 cells, or MOLT‐4 cells on 12‐mm^2^ cover slips. A noncontact culture between HCT‐5 cells/MOLT‐4 cells and FDC‐like cells for the ELISAs was performed by using a Transwell® Permeable Support with a 0.4‐mm polyester membrane (Corning). Immobilized antigens including BAFF and CXCL13 were subjected to antigen‐antibody reaction with a human BAFF/BLyS/TNFSF13B or human CXCL13/BLC/BCA‐1 Quantikine ELISA kit (R&D Systems). We used 50 μl of assay diluents as single samples, positive human serum as a positive control, and normal human serum as a negative control.

The samples were added to the wells of a microplate on which an immobilized monoclonal antibody specific for BAFF/BLyS/TNFSF13B or CXCL13/BLC/BCA‐1 was precoated. After the wells were washed with wash buffer, the reaction was incubated with peroxidase‐conjugated anti‐human immunoglobulin polyclonal antibody as a secondary reaction, followed by the measurement of A_450_ absorbance.

### 2.8 Statistical analysis

We used Welch's *t* test to examine the results of the GMFI by FCM, the MFI by IF, and the ELISAs. The *p* values of less than .05 were considered significant.

## 3 RESULTS

### 3.1 Establishment of FDC‐like cells

**Figure 1:**
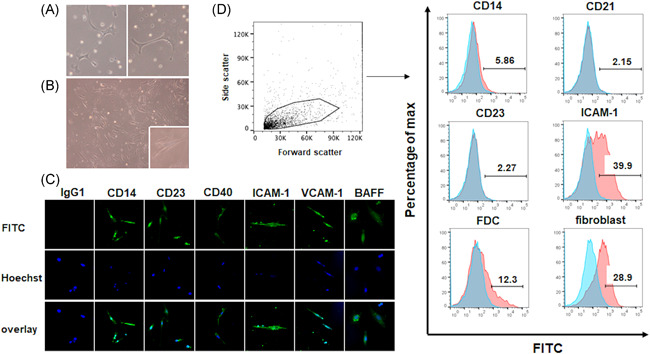
Morphological characteristics of the FDC‐like cells at an early phase. (A) The morphological appearance of FDC‐like cells on a 100‐mm^2^ dish on Day 2 of culture. (B) The change of morphological characteristics on Day 7. Original magnifications, (A): ×200, (B): ×40. Inset in panel B is a magnified view of Day‐7 FDC‐like cells. (C) Expressions of CD14, CD23, CD40, ICAM‐1, VCAM‐1, and BAFF on Day 2 were examined by immunofluorescence by using FITC‐conjugated primary antibodies. (D) To detect markers on FDC‐like cells at the established phase, a flow cytometry (FCM) analysis after the gating of FDC‐like cells was performed for CD14, CD21, CD23, ICAM‐1, FDC, and fibroblast cell marker. FITC‐conjugated mouse IgG1 was used as an internal control. Representative results of five independent experiments with similar results are shown. BAFF, B‐cell activating factor; FDC, follicular dendritic cell; ICAM‐1, intracellular adhesion molecule‐1; VCAM‐1, vascular cell adhesion molecule‐1

The attached cells in Day‐2 cultures showed morphologically dendritic‐like features (Figure 1A). After 1 week of culture, the morphology had changed to a fibroblastic figuration (Figure 1B). IF on Day 2 showed the expressions of CD14, CD23, CD40, ICAM‐1, VCAM‐1, and BAFF (Figure 1C). Based on the gating of FDC‐like cells, the established cell line expressed ICAM‐1, FDC, and fibroblast cell marker, and the expressions of ICAM‐1 and fibroblast cell marker were especially clear (Figure 1D and Table 1). In contrast, no expression of CD21 or CD23 was observed. Table 1 summarizes the expressions of the molecules. The cell viability was more than 95% when the percentage of live cells was calculated with the Zombie Aqua™ fixable viability kit.

**Table 1:** Comparison of expressed markers on FDC‐like cells

Markers in IF at Day 2		Markers in FCM	
CD14	positive	CD14	slightly positive
CD23	positive	CD21	negative
CD40	positive	CD23	negative
ICAM‐1	positive	ICAM‐1	positive
VCAM‐1	positive	FDC	positive
BAFF	positive	Fibroblast	positive

Abbreviations: BAFF, B‐cell activating factor belonging to the tumor necrosis factor family; FCM, flow cytometry; FDC, follicular dendritic cell; ICAM‐1, intracellular adhesion molecule‐1; IF, immunofluorescence; VCAM‐1, vascular cell adhesion molecule‐1.

### 3.2 BAFF expression by unstimulated and stimulated FDC‐like cells

**Figure 2:**
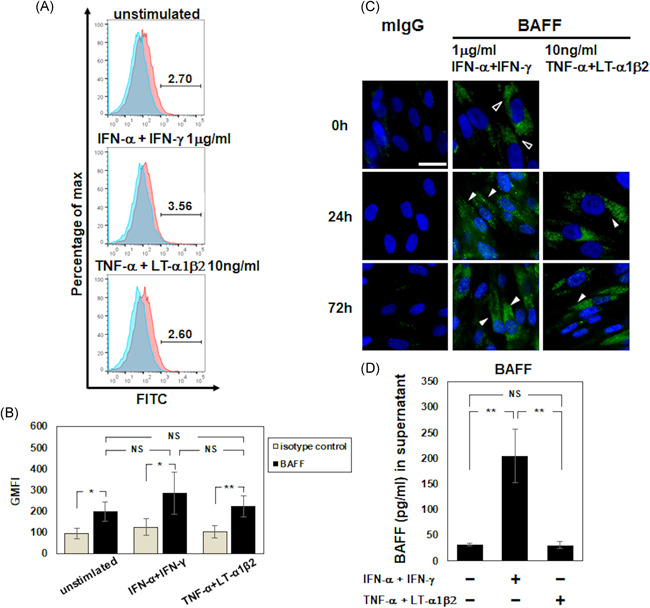
Expression of BAFF on FDC‐like cells. (A) The expression of BAFF on unstimulated FDC‐like cells at a continuously cultivated phase shown by FCM. (B) Geometric mean fluorescence intensity (GMFI) of BAFF on the FDC‐like cells by FCM in the presence/absence of 1 μg/ml of IFN‐α/IFN‐γ or 10 ng/ml of TNF‐α/LTα1β2. (C) The expression of BAFF was examined at 0, 24, and 72 h by IF in the presence and absence of 1 μg/ml of IFN‐α/IFN‐γ or 10 ng/ml of TNF‐α/LTα1β2. Mouse IgG1 was used as an internal control. Bar: 20 μm. The open arrowheads show the BAFF expression without stimulation, and the filled arrowheads show the expression after stimulation. (D) The concentration of BAFF was measured by an ELISA in the presence/absence of 1 μg/ml of IFN‐α/IFN‐γ or 10 ng/ml of TNF‐α/LTα1β2. Representative results of four independent experiments with similar results are shown. Data are mean (*SD*). *p < .05, **p < .01 by Welch's *t* test. NS: not significant. BAFF, B‐cell activating factor; ELISA, enzyme‐linked immunosorbent assay; FDC, follicular dendritic cell; IF, immunofluorescence; IFN, interferon; IgG, immunoglobulin G; LT: lymphotoxin; TNF, tumor necrosis factor

The expression of surface BAFF was tested by FCM in the presence/absence of 1 μg/ml of IFN‐α and IFN‐γ. The results revealed slight BAFF expression on the surface of unstimulated FDC‐like cells, and no augmentation of BAFF was observed regardless of the presence of these IFNs (Figure 2A,B). Similarly, treatment with 10 ng/ml of TNF‐α and LTα1β2 produced no augmentation of surface BAFF expression at 48 h of stimulation.

BAFF expression was also tested by IF in the presence/absence of 1 μg/ml of IFN‐α/IFN‐γ or 10 ng/ml of TNF‐α/LTα1β2; the results demonstrated the constitutive expression of cytoplasmic BAFF at 0–72 h regardless of the presence/absence of these cytokines (Figure 2C). As shown in Figure 2D, 24‐HR stimulation with 1 μg/ml of IFN‐α/IFN‐γ but not 10 ng/ml of TNF‐α/LTα1β2 significantly augmented the BAFF concentration (*p* < .01).

### 3.3 CXCL13 expression by unstimulated and stimulated FDC‐like cells

**Figure 3:**
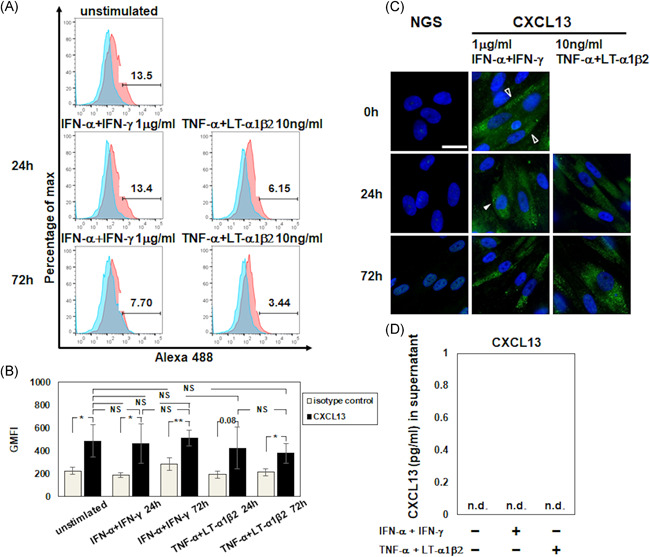
Expression of CXCL13 on FDC‐like cells. (A) The expression of CXCL13 on unstimulated FDC‐like cells at a continuously cultivated phase shown by FCM. (B) The GMFI of CXCL13 on the FDC‐like cells by FCM in the presence/absence of 1 μg/ml of IFN‐α/IFN‐γ or 10 ng/ml of TNF‐α/LTα1β2. (C) The expression of CXCL13 was examined at 0, 24, and 72 h by IF in the presence/absence of 1 μg/ml of IFN‐α/IFN‐γ or 10 ng/ml of TNF‐α/LTα1β2. Normal goat serum (NGS) was used as an internal control. Bar: 20 μm. The open arrowheads show the CXCL13 expression without stimulation, and the filled arrowheads show the expression after stimulation. (D) The concentration of CXCL13 was measured by an ELISA in the presence/absence of 1 μg/ml of IFN‐α/IFN‐γ or 10 ng/ml of TNF‐α/LTα1β2. Representative results of four independent experiments with similar results are shown. Data are mean (*SD*). *p < .05, **p < .01 by Welch's *t* test. NS: not significant. n.d.: not detected. CXCL13: C‐X‐C motif chemokine ligand 13. BAFF, B‐cell activating factor; CXCL, C‐X‐C motif ligand; FDC, follicular dendritic cell; GMFI, geometric mean fluorescence intensity; IFN, interferon; IgG, immunoglobulin G; LT: lymphotoxin; TNF, tumor necrosis factor

Slight CXCL13 expression was observed in unstimulated FDC‐like cells (Figure 3A,B). The CXCL13 expression was not augmented by the stimulation of FDC‐like cells with 1 μg/ml of IFN‐α/IFN‐γ or 10 ng/ml of LTα1β2/TNF‐α for 24 or 72 h (Figure 3A,B). The CXCL13 expression was also evaluated by IF in the presence/absence of 1 μg/ml of IFN‐α/IFN‐γ or 10 ng/ml of TNF‐α/LTα1β2; the results showed the constitutive expression of cytoplasmic CXCL13 at 0–72 h regardless of the presence/absence of these cytokines (Figure 3C). No augmentation of the CXCL13 concentration was observed after 24‐h stimulation with 1 μg/ml of IFN‐α/IFN‐γ or with 10 ng/ml of TNF‐α/LTα1β2 (Figure 3D).

### 3.4 Decreased BAFF and CXCL13 expressions by direct coculture with HTLV‐1 infected cell lines shown by IF

**Figure 4:**
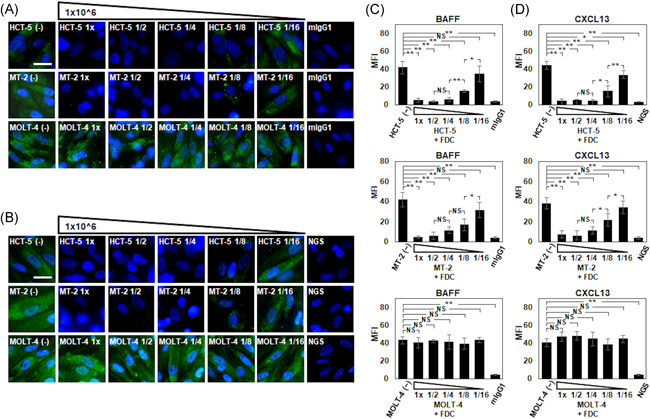
The effect of HTLV‐1‐infected cell lines on the expressions of BAFF and CXCL13 on FDC‐like cells. (A) The expression of BAFF under the presence of HCT‐5 cells, MT‐2 cells, or MOLT‐4 cells for 24 h is shown by IF according to the double‐fold diluted counts of HCT‐5 cells, MT‐2 cells, or MOLT‐4 cells. BAFF was stained with mouse anti‐BAFF monoclonal antibody followed by FITC‐conjugated anti‐mouse secondary antibody. Mouse IgG (mIgG): Internal control. (B) The expression of CXCL13 under the presence of HCT‐5 cells, MT‐2 cells, or MOLT‐4 cells for 24 h is shown by IF according to the double‐fold diluted counts of HCT‐5 cells, MT‐2 cells, or MOLT‐4 cells. CXCL13 was stained with goat anti‐CXCL13 polyclonal antibody followed by FITC‐conjugated anti‐goat secondary antibody. Normal goat serum (NGS): Internal control. Bar: 10 μm. (C) The mean fluorescence intensity (MFI) of BAFF on the FDC‐like cells by IF. (D) The MFI of CXCL13 on the FDC‐like cells by IF. Representative results of four independent experiments with similar results are shown. Data are mean (*SD*). *p < .05, **p < .01 by Welch's *t* test. BAFF, B‐cell activating factor; CXCL, C‐X‐C motif ligand; FDC, follicular dendritic cell; HTLV‐1, human T‐cell leukemia virus type 1; IgG, immunoglobulin G

Before we conducted the coculture experiments, we observed that CXCR5 was slightly and Gag was strongly expressed on unstimulated HCT‐5 cells and MT‐2 cells, but they were not expressed on MOLT‐4 cells (Figure S1). The BAFF expression by unstimulated FDC‐like cells was clearly suppressed by direct coculture with 1 × 10^6^ HCT‐5 cells/ml or 1 × 10^6^ MT‐2 cells/ml (Figures 4A and 4C). The BAFF expression gradually recovered in accord with the decreased ratio (1× to 1/16) of HCT‐5 cells or MT‐2 cells. The CXCL13 expression by unstimulated FDC‐like cells was also clearly suppressed by direct coculture with 1 × 10^6^ HCT‐5 cells/ml or 1 × 10^6^ MT‐2 cells/ml (Figures 4B and 4D). The CXCL13 expression gradually recovered in accord with the decreased ratio of HCT‐5 cells or MT‐2 cells. In contrast, no suppression of BAFF or CXCL13 was observed when FDC‐like cells were cocultured with MOLT‐4 cells.

**Figure 5:**
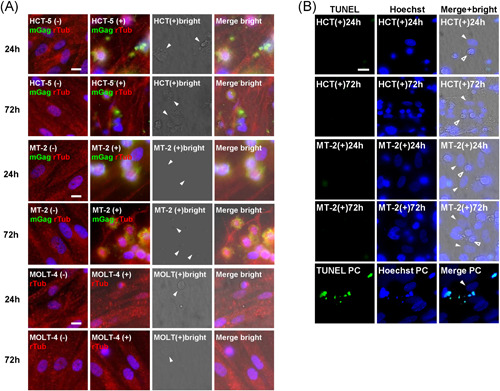
The evaluation of Gag expression and apoptosis of FDC‐like cells after coculture with HTLV‐1‐infected cell lines. (A) Morphological change of FDC‐like cells after 24‐ and 72‐h coculture with HCT‐5 cells, MT‐2 cells, or MOLT‐4 cells. HCT‐5 cells and MT‐2 cells were stained with mouse anti‐HTLV‐1‐Gag monoclonal antibody followed by FITC‐conjugated anti‐mouse secondary antibody. Rabbit antialpha‐tubulin antibody followed by rhodamine‐conjugated anti‐rabbit secondary antibody (red) was used for tracing the FDC‐like cell morphology. The bright‐field view was used to confirm the existence of HCT‐5 cells, MT‐2 cells, or MOLT‐4 cells (arrowheads). (B) Apoptosis determined by TUNEL staining at 24 and 72 h. The bright‐field view was used to distinguish FDC‐like cells (arrowheads) from HCT‐5 cells (open arrowheads). As a positive control of apoptosis, the induction of apoptosis of FDC‐like cells was accomplished with 10 μM actinomycin D, 5.7 μM camptothecin, 100 μM cycloheximide, 10 μM dexamethasone, and 100 μM etoposide. Bar: 10 μm. Representative results of four independent experiments with similar results are shown. FDC, follicular dendritic cell; GMFI, geometric mean fluorescence intensity; HTLV‐1, human T‐cell leukemia virus type 1

After 24 and 72‐h direct cocultures of FDC‐like cells with HCT‐5 cells, MT‐2 cells, or MOLT‐4 cells, no obvious infection as merged signals was detected on the FDC‐like cells (Figure 5A). However, a styliform change of FDC‐like cells was observed (Figure 5A) after 24‐ and 72‐h cocultures with HCT‐5 cells or MT‐2 cells; this change was not observed in the coculture with MOLT‐4 cells. The intracellular expressions of BAFF and CXCL13 were constitutively observed at 24‐ and 72‐h direct cocultures regardless of the use/nonuse of IFNs or TNF/LT, but the expressions of BAFF and CXCL13 were clearly suppressed by coculturing with HCT‐5 cells (Figure S2A,B). There was no augmentation of the MFI of BAFF and CXCL13 by the addition of IFNs or TNF/LT in each coculture/noncoculture (Figure S3A,B). However, the overall MFI values of BAFF and CXCL13 in coculture with HCT‐5 cells were low. Faint BAFF expression and clear CXCL13 expression by a small number of HCT‐5 cells among HCT‐5 cell aggregates were observed (Figure S2C).

Although a styliform change was observed after 24‐ and 72‐h cocultures with HCT‐5 cells or MT‐2 cells, no apoptosis was detected on FDC‐like cells by TUNEL staining. The positive control using an apoptosis inducer showed typical apoptosis (Figure 5B).

### 3.5 Different expressions of BAFF and CXCL13 in culture supernatant

**Figure 6:**
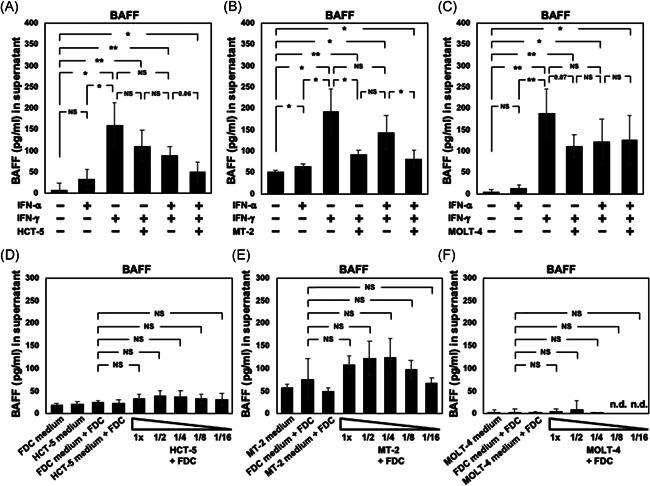
The expressions of BAFF in culture supernatant. (A–C) The concentrations of BAFF in FDC‐like cell culture medium were measured after stimulation with 1 μg/ml of interferon (IFN)‐α, IFN‐γ, or both for 48 h. The concentrations of these molecules with 1 μg/ml of IFN‐α and IFN‐γ under the presence of 1 × 10^6^ HCT‐5 cells/ml (A), MT‐2 cells/ml (B), or MOLT‐4 cells/ml (C) were measured. (D–F) The expression of BAFF in supernatant from FDC‐like cells in the absence or presence of various concentrations of HCT‐5 (D), MT‐2 (E), or MOLT‐4 (F) was examined by ELISA. The samples of cocultured medium were centrifuged at 10,000 rpm for 5 min at 4℃, and then the supernatant was collected in new tubes. FDC culture medium and HCT‐5/MT‐2/MOLT‐4 culture medium were also measured as internal controls. Data are mean ± *SD* from four independent experiments. *p < .05, **p < .01 by Welch's *t* test. BAFF, B‐cell activating factor; ELISA, enzyme‐linked immunosorbent assay; FDC, follicular dendritic cell

The BAFF concentration in the FDC‐like cell culture medium was elevated by stimulation with 1 μg/ml of IFN‐α, IFN‐γ, or both. IFN‐γ treatment clearly augmented the BAFF expression. The coculture with 1 × 10^6^ HCT‐5 cells/ml under the presence of IFN‐γ showed a reduced BAFF concentration, without significance (Figure 6A). Compared to the BAFF concentration stimulated with IFN‐α and IFN‐γ, the addition of HCT‐5 cells resulted in a decreased concentration of BAFF (*p* = .06). The addition of MT‐2 cells significantly reduced the concentration of BAFF under the stimulation with IFN‐γ or IFN‐α/γ (Figure 6B). In contrast, the addition of MOLT‐4 cells showed no reduction of the BAFF concentration that was produced by stimulation with IFN‐α and IFN‐γ, although we observed a decrease of the BAFF concentration when MOLT‐4 cells were stimulated with IFN‐γ (*p* = .07) (Figure 6C).

The concentration of BAFF was not changed by the addition of 1 × 10^6^ HCT‐5, MT‐2, or MOLT‐4 cells/ml without stimulation with IFNs, and no significant change in the concentration was observed regardless of the number of HCT‐5 cells (Figure 6D), MT‐2 cells (Figure 6E), or MOLT‐4 cells (Figure 6F) except for the difference between 1 × 10^6^ MT‐2 cells/ml and 1/16 × 10^6^ of MT‐2 cells (*p* = .013).

**Figure 7:**
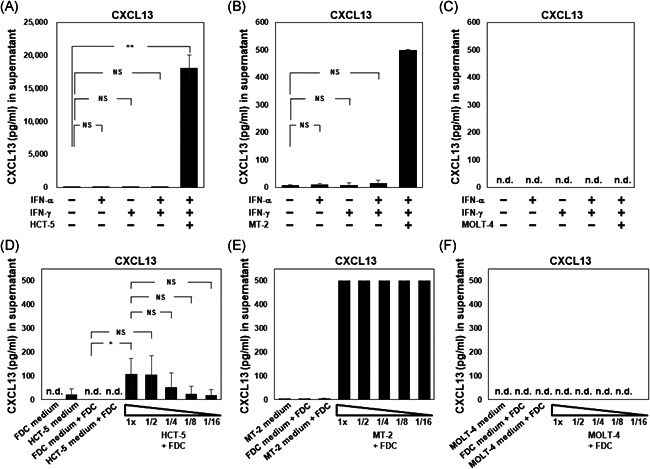
The expressions of CXCL13 in culture supernatant by ELISA. (A–C) The concentrations of CXCL13 in FDC‐like cell culture medium were measured after stimulation with 1 μg/ml of IFN‐α, IFN‐γ, or both for 48 h. The concentrations of these molecules with 1 μg/ml of IFN‐α and IFN‐γ under the presence of 1 × 10^6^ HCT‐5 cells/ml (A), MT‐2 cells/ml (B), or MOLT‐4 cells/ml (C) were measured. (A) The mean ± *SD* of CXCL13 concentrations in supernatants of unstimulated FDC‐like cells, IFN‐α‐stimulated FDC‐like cells, IFN‐γ‐stimulated FDC‐like cells, and IFN‐α/γ‐stimulated FDC‐like cells were 6.6 ± 4.61, 4.6 ± 5.64, 2.4 ± 2.77, and 1.8 ± 3.58, respectively. (D–F) The expression of CXCL13 in supernatant from FDC‐like cells in the absence or presence of various concentrations of HCT‐5 (D), MT‐2 (E), or MOLT‐4 (F) cells was examined by ELISA. The samples of cocultured medium were centrifuged at 10,000 rpm for 5 min at 4℃, and then the supernatant was collected in new tubes. FDC culture medium and HCT‐5/MT‐2/MOLT‐4 culture medium were also measured as internal controls. Data are mean ± *SD* from four independent experiments. *p < .05, **p < .01 by Welch's *t* test. CXCL, C‐X‐C motif ligand; ELISA, enzyme‐linked immunosorbent assay; FDC, follicular dendritic cell; IFN, interferon

The CXCL13 concentration was not changed by any stimulation, but it was high under the presence of HCT‐5 cells or MT‐2 cells (Figure 7A,B), and CXCL13 was not detected when MOLT‐4 cells were added under the presence of IFN‐α and/or IFN‐γ (Figure 7C). The concentration of CXCL13 was high with the addition of 1 × 10^6^ HCT‐5 cells/ml, and the concentration was gradually decreased depending on the number of HCT‐5 cells (Figure 7D). The CXCL13 concentration was more than 500 pg/ml under the presence of MT‐2 cells (Figure 7E), although CXCL13 was not detected when different concentrations of MOLT‐4 cells were added to FDCs (Figure 7F).

Similarly, the concentration of CXCL13 (but not that of BAFF) in the supernatant was increased by indirect coculturing with an increased number of HCT‐5 cells (Figure S4A,B). Although BAFF was not detected in the culture supernatant of HCT‐5 cells, the CXCL13 concentration in this supernatant was extremely high (16643 ± 165 pg/ml) (data not shown).

Morphological changes of FDC‐like cells were observed under the presence of HCT‐5 cells but not the IFNs (Figure S4C). The addition of MOLT‐4 cells showed no morphological changes to FDC‐like cells. Similarly, a morphological change was observed when HCT‐5 cells were directly added (Figure S4D), but there was no morphological change in the case of indirect coculture. Interestingly, the styliform change of the FDC‐like cells in direct coculture was recovered by a decreased percentage of HCT‐5 cells (Figure S4D). The culture of FDC‐like cells in HCT‐5 culture medium (20% FBS/RPMI with IL‐2) resulted in no morphological changes, regardless of the direct or indirect addition of this culture medium. The addition of different concentrations of MOLT‐4 cells resulted in no morphological changes to FDC‐like cells.

### 3.6 Sera from the SS patients

**Figure 8:**
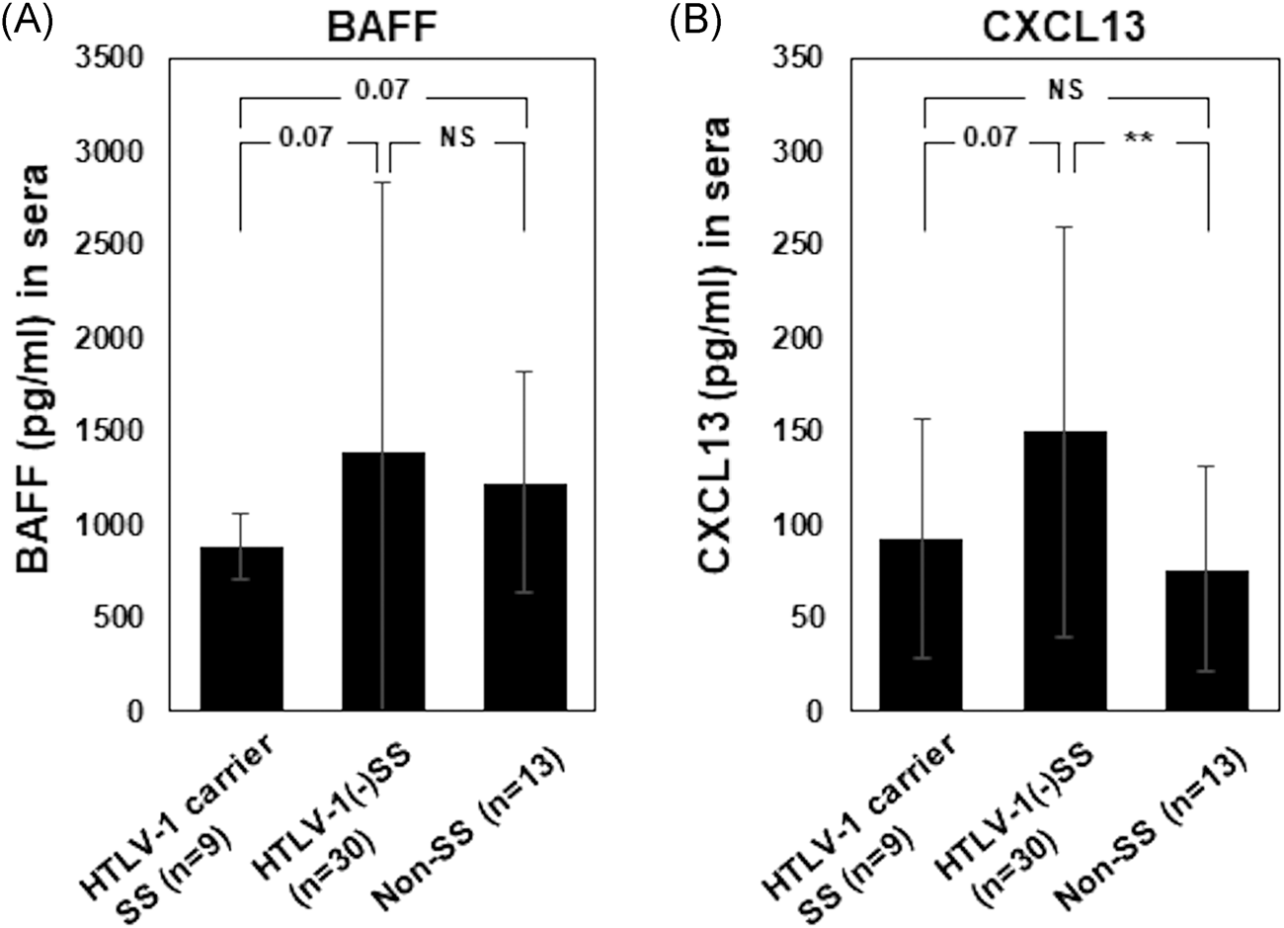
The expressions of BAFF and CXCL13 in sera from HTLV‐1‐seropositive subjects. The expressions of BAFF (A) and CXCL13 (B) in sera from HTLV‐1 carriers with SS (*n* = 9), HTLV‐1‐seronegative SS patients (*n* = 30), and non‐SS subjects (*n* = 13) were examined by ELISAs. Data are mean (SD). **p < .01 by Welch's *t* test. BAFF, B‐cell activating factor; CXCL, C‐X‐C motif ligand; HTLV‐1, human T‐cell leukemia virus type 1

The serum BAFF level of the HTLV‐1 carriers with SS was lower than that of the HTLV‐1‐seronegative SS and non‐SS subjects (*p* = .07) (Figure 8A). The serum CXCL13 level was increased in the HTLV‐1‐seronegative SS group compared to the non‐SS subjects, although the serum CXCL13 level of the HTLV‐1 carriers with SS was lower than that of the HTLV‐1‐seronegative SS patients (*p* = .07) (Figure 8B).

## 4 DISCUSSION

We established 22 FDC‐like cells that express cell surface markers, BAFF, and CXCL13. The constitutive expressions of BAFF and CXCL13 were confirmed by intracellular staining by IF. The cytoplasmic BAFF and CXCL13 expressions on FDC‐like cells were significantly decreased by coculturing with two HTLV‐1‐positive cell lines, HCT‐5 and MT‐2. Direct (but not indirect) coculture with HCT‐5 and MT‐2 cells inhibited the expressions of BAFF and CXCL13 with an elongated change of FDC‐like cells. The increase in the BAFF concentration by the addition of IFNs in culture supernatant showed a reduced tendency under the presence of HCT‐5 and MT‐2 cells. Since the HCT‐5 and MT‐2 cells did not alter the unstimulated secreted BAFF concentration, these two cell lines had the potential to inhibit the IFN‐mediated BAFF secretion.

We also observed the CXCL13 concentration was high under the presence of HCT‐5 cells, and the CXCL13 concentration increased in accord with the HCT‐5 cell count in the direct coculture samples' supernatant. In addition, the serum concentrations of both BAFF and CXCL13 in the HTLV‐1‐seropositive SS patients were lower than those of the HTLV‐1‐seronegative SS patients.

The morphological findings and characteristic markers of FDCs are fundamentally similar to those of mesenchymal stem cells.^25^ It was originally reported that FDC‐associated antigens including CD14, CD21, CD23, BAFF, and CXCL13 were autonomously observed.^24^ However, Kim et al.^26^ reported characteristics of FDC‐like cells; they noted that HK cells (an FDC‐like cell line) have morphologically cytoplasmic extensions at an early stage of cell culture. They also observed that CD21 and CD23 disappeared after 3 days of cell culture. Although these findings differ from those reported for the original FDCs,^24^ the expression patterns of surface markers including the disappearance of CD21 and CD23 observed on HK cells were similar to those on the FDC‐like cells examined herein.

Regarding the association between HTLV‐1 infection and autoimmune diseases, we reported a low prevalence of anti‐Ro/SS‐A antibody in HTLV‐1‐seropositive SS patients.^16^, ^17^ A unique characteristic, that is, low prevalences of autoantibodies, was observed particularly in the HAM patients with SS. The differing clinical findings between HTLV‐1 carriers and HAM patients might be explained in part by the high proviral load in the HAM patients' sera,^27^ and our additional investigation revealed that the LSGs of patients with HAM‐SS had significantly low numbers of eGCs.

Because eGCs contains FDCs in the outer lining,^28^ the interaction of FDCs and B cells is crucial in the autoantibody production system.^29^, ^30^ We should also note that the selection of high‐affinity B cells is closely associated with the presence of Tfh cells. For example, Liu et al.^31^ reported that inducible T‐cell costimulator ligand supported a Tfh‐B‐cell interaction in the GC toward the production and maintenance of high‐affinity plasma cells. Our present analyses demonstrated significantly decreased BAFF and CXCL13 expressions under the presence of HCT‐5 and MT‐2 cells, indicating that HTLV‐1‐infected cells might directly inhibit the function of FDC‐like cells in accord with the induction of styliform change, although no apoptosis was detected. The question of whether HTLV‐1 infection has one or more direct effects on B cells or plasma cells that have the potential to produce immunoglobulin or specific autoantibodies remains to be investigated.

Although we have found no report regarding the inhibition of BAFF or CXCL13 in the presence of HTLV‐1‐infected cells, HCT‐5 and MT‐2 cells might have the direct effect of inhibiting these chemokines, accompanied by the altered morphology of FDC‐like cells. Because our previous findings showed the disappearance of both the eGCs and the CXCL13 expression in LSGs, the inhibitory effect of HCT‐5 and MT‐2 cells on FDC‐like cells observed herein is a novel finding that may help explain why the LSGs of HAM‐SS patients show a hypoplastic formation of eGCs compared to the LSGs of HTLV‐1‐seronegative patients with SS.

Although BAFF is essential for focal autoantibody production, the cells that produce BAFF include various types of immune cells such as peripheral monocytes, macrophages,^32^ dendritic cells, and activated T cells.^33^ Moore et al.^32^ showed that the stimulation of monocytes with IFN‐γ upregulated BLyS/BAFF expression, and the IFN‐γ‐mediated augmentation of the BAFF level in our present investigation is consistent with this finding.

Although a reduction of the BAFF concentration stimulated with both IFN‐α and IFN‐γ was observed under the presence of HCT‐5 and MT‐2 cells, the declining tendency of the BAFF concentration was also observed when FDC‐like cells stimulated with IFN‐γ were cocultured with MOLT‐4 cells. There were no morphological changes when FDC‐like cells were directly cocultured with MOLT‐4 cells, but there might be mechanical stress for FDC‐like cells regardless of the contacting cell species. Regarding HTLV‐1‐related gene and protein expression, we showed^34^ high expressions of tax and HTLV‐1 b ZIP factor in HCT‐5 cells in a comparison with HTLV‐1‐seropositive cell lines derived from ATL patients. We also demonstrated that HCT‐5 cells had HTLV‐1 virions on the surface by immunoelectron microscopy.^35^ Infection with FDC could not be confirmed in Figure 4A, But since the release of HTLV‐1 virions from HCT‐5 cells was confirmed by electron microscopy,^35^ it is possible that the virus on HCT‐5 cells or the released virus particles might be associated with a suppression of cytokines. An indirect culture using Transwells did not show any decrease in BAFF levels and morphologically no change in FDC‐like cells. These results suggested that direct contact was important.

We unexpectedly detected an elevated CXCL13 concentration in the HCT‐5 culture supernatant. A CXCL13‐producing CD4 T‐cell subset was reported in rheumatoid arthritis.^36^ We measured the CXCL13 concentration in the supernatant of MT‐2 cells that were derived from an ATL patient. Because the concentration of MT‐2 cells as well as that of HCT‐5 cells was high, the secretion of CXCL13 might be a characteristic of HTLV‐1 infected cells. As there is apparently no previous report regarding elevated CXCL13 in HTLV‐1‐infected cells, our present findings may constitute novel evidence concerning the pathogenesis of HAM.

Regarding the serum concentrations of BAFF and CXCL13, the low BAFF level in the HTLV‐1‐seropositive SS patients might reflect an HTLV‐1‐related condition, although there are no references to confirm these phenomena. In contrast, we observed that the serum CXCL13 level in the HTLV‐1‐seronegative SS patients was higher than that of the non‐SS subjects, as reported.^37^ The lower serum CXCL13 level of the HTLV‐1‐seropositive SS patients compared to that of the HTLV‐1‐seronegative SS patients might indicate differences in focal inflammation in salivary glands in both groups. The serum BAFF levels tended to be higher for the anti‐HTLV‐1 antibody‐negative SS patients, which was consistent with our in vitro results. However, the BAFF concentration in the non‐SS subjects was similar to that in the HTLV‐1 antibody‐negative SS patients. Since the BAFF concentration varied widely in the HTLV‐1‐negative SS cases, it is possible that the BAFF expression varied depending on the disease activity of SS. For this reason, we speculate that the serum BAFF concentration might not be different from non‐SS.

On the other hand, we observed that the CXCL13 concentration was lower in the HTLV‐1 carrier SS cases. CXCL13 was elevated in the HCT‐5 and MT‐2 supernatants, and CXCL13 may vary depending on the amount of HTLV‐1 virus. It will be informative to determine the serum BAFF and CXCL13 concentrations in HTLV‐1 carriers in SS and non‐SS subjects. Because there was no non‐SS subject with anti‐HTLV‐1 antibody in our present study, we plan to accumulate these cases in the future.

Taken together, our findings demonstrate newly established FDC‐like cells and their BAFF/CXCL13 expression pattern. We also observed that HTLV‐1 infection directly influenced the FDC‐like cells' morphology and the expressions of chemokines that are important for B‐cell activation, suggesting that these findings can explain in part the low frequency of autoantibodies in HAM‐SS patients. We plan to investigate the direct effect of HTLV‐1 infection on the production of autoantibodies including anti‐Ro/SS‐A and La/SS‐B antibodies. Toward this end, we will attempt to construct a system showing that HTLV‐1 infection can infect B cells or plasma cells and subsequently inhibit B cells' functions.

## CONFLICT OF INTERESTS

The authors declare that there are no conflict of interests.

## AUTHOR CONTRIBUTIONS

**Hideki Nakamura and Ayuko Takatani**: study conception and design. **Kyoko Kitaoka**: tonsil biopsy. **Ayuko Takatani, Yushiro Endo, Masataka Umeda, Hideki Nakamura, and Kaori Furukawa**: acquisition of data. **Hideki Nakamura, Toshimasa Shimizu, Shin‐ya Nishihata, Tatsufumi Nakamura, and Atsushi Kawakami**: analysis and interpretation of data. **Ayuko Takatani and Hideki Nakamura**: equally contributed to this work. All authors were involved in drafting the article or revising it critically for important intellectual content, and all authors approved the final version for publication.

## Supporting information

Supporting information.

Supporting information.

Supporting information.

Supporting information.

Supporting information.

Supporting information.

## Data Availability

The datasets during and/or analysed during the current study available from the corresponding author on reasonable request. H. Nakamura has full access to all of the data in the study and takes responsibility for the integrity of the data and the accuracy of the data analysis. All data analyzed in this study are included in this article.
