# Supplementary figures and images for: Inhibitory effect of HTLV‐1 infection on the production of B‐cell activating factors in established follicular dendritic cell‐like cells
Source: Immun Inflamm Dis. 2021 May 4;9(3):777–91. doi: 10.1002/iid3.432 (PMC8342235; doi:10.1002/iid3.432)

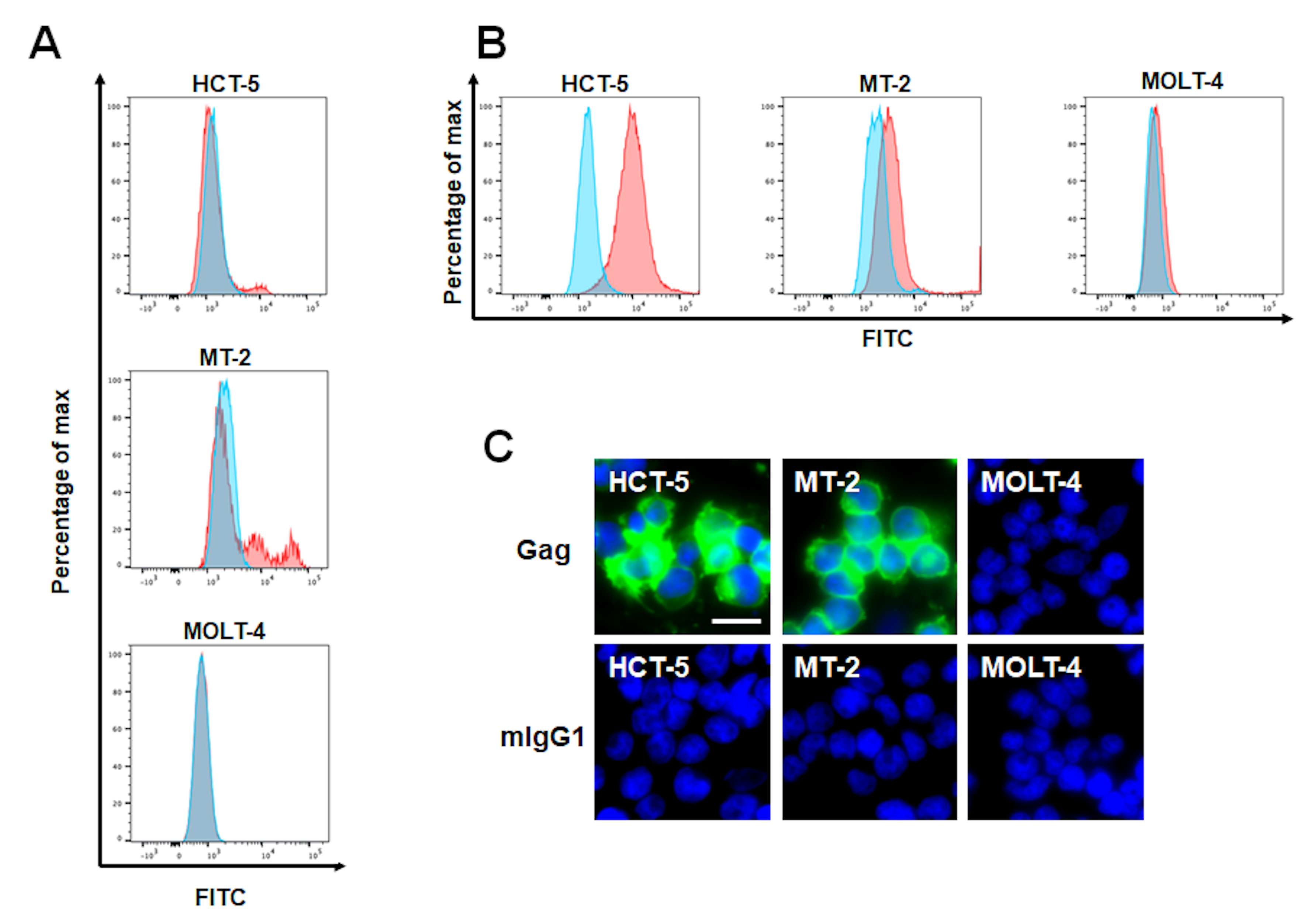

Supplement: Supplementary file 1 — Supporting information. [file IID3-9-777-s002.png]

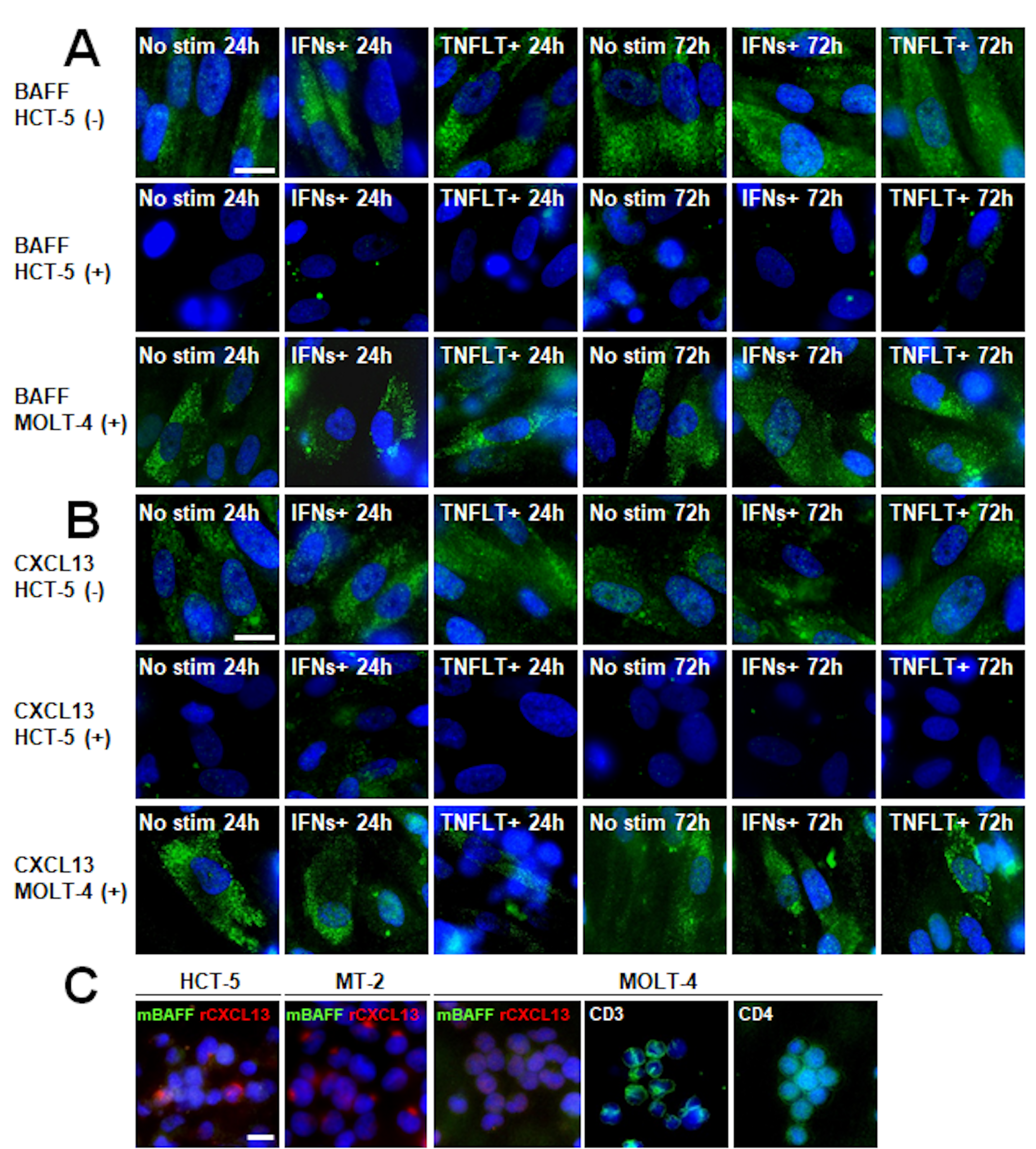

Supplement: Supplementary file 2 — Supporting information. [file IID3-9-777-s003.png]

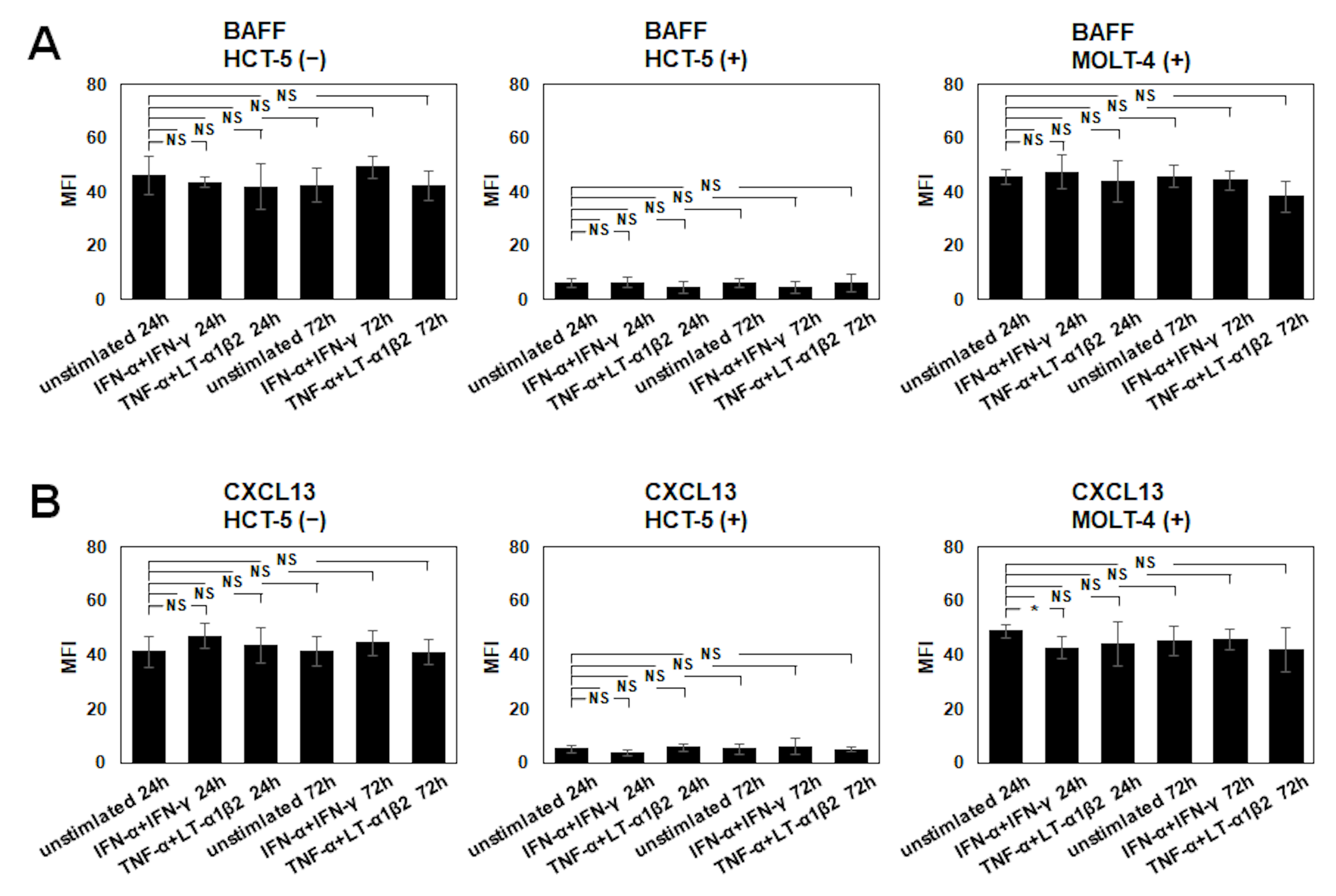

Supplement: Supplementary file 3 — Supporting information. [file IID3-9-777-s004.png]

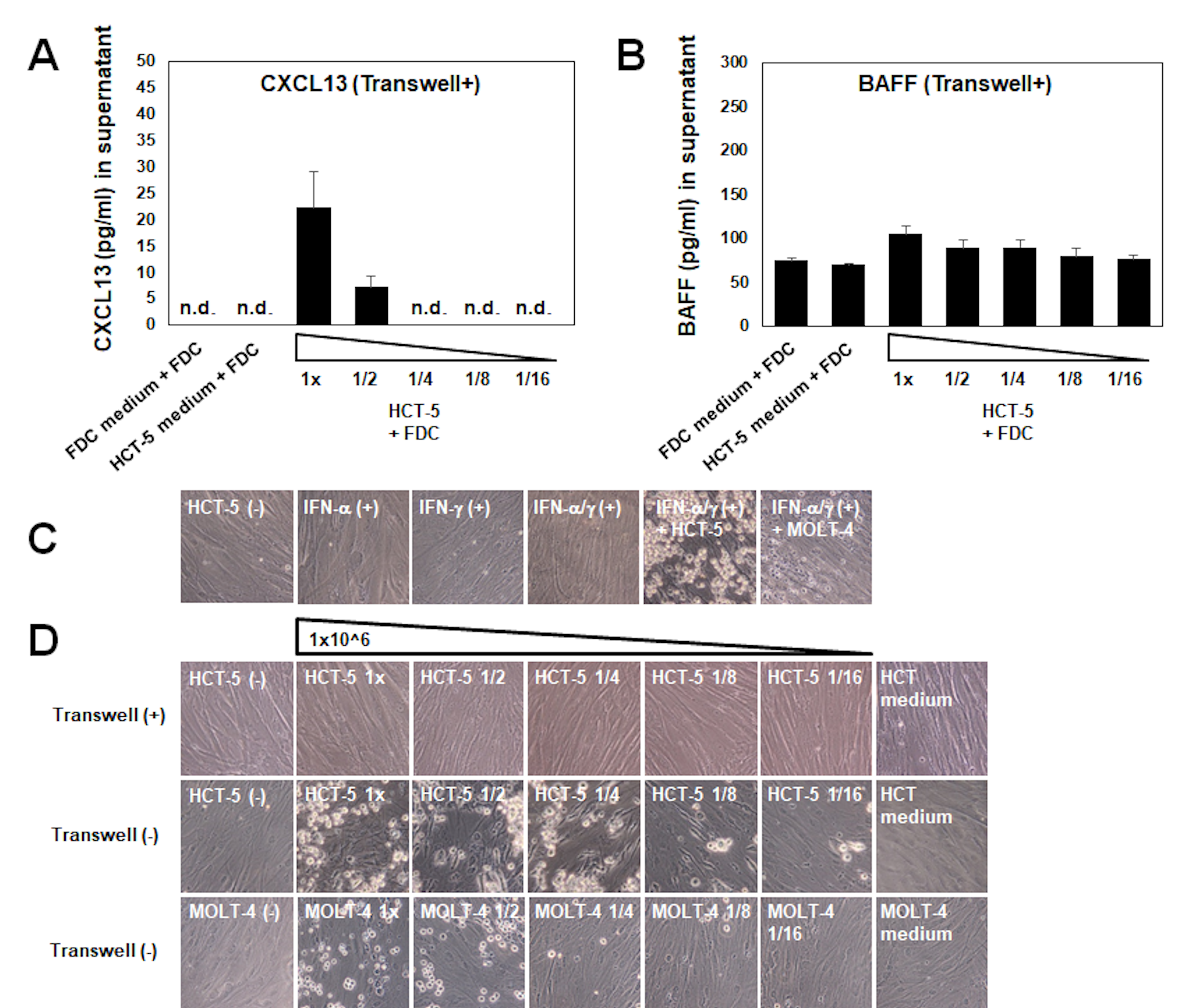

Supplement: Supplementary file 4 — Supporting information. [file IID3-9-777-s005.png]
