# Supplementary material for: Inhibitory effect of HTLV‐1 infection on the production of B‐cell activating factors in established follicular dendritic cell‐like cells
Source: Immun Inflamm Dis. 2021 May 4;9(3):777–91. doi: 10.1002/iid3.432 (PMC8342235; doi:10.1002/iid3.432)
Supplement: Supplementary file 5 — Supporting information. [file IID3-9-777-s006.doc]

**Supplementary Table S1. Primary antibodies used in this study**

| **Antibody** | **Mono/poly** | **Species** | **Application** | **Labeling** | **Dilution** |
| --- | --- | --- | --- | --- | --- |
| CD14 | monoclonal | mouse | IF | No | 1:100 |
| CD23 | monoclonal | mouse | IF | No | 1:100 |
| CD40 | monoclonal | mouse | IF | No | 1:100 |
| ICAM-1 | monoclonal | mouse | IF | No | 1:100 |
| VCAM-1 | monoclonal | mouse | IF | No | 1:100 |
| BAFF | monoclonal | mouse | IF | No | 1:100 |
| CXCL13 | polyclonal | goat | IF | No | 1:100 |
| CD14 | monoclonal | mouse | FCM | FITC | 1:20 |
| CD21 | monoclonal | mouse | FCM | FITC | 1:20 |
| CD23 | monoclonal | mouse | FCM | FITC | 1:20 |
| ICAM-1 | monoclonal | mouse | FCM | FITC | 1:20 |
| FDC (CNA42) | monoclonal | mouse | FCM | No | 1:20 |
| fibroblast | monoclonal | mouse | FCM | No | 1:20 |
| BAFF | monoclonal | mouse | FCM | FITC | 1:20 |
| CXCL13 | monoclonal | mouse | FCM | Alexa Fluor488 | 1:20 |
| CXCR5 | monoclonal | mouse | FCM | No | 1:100 |
| HTLV-1 (Gag) | monoclonal | mouse | IF | No | 1:100 |
| Alpha-tubulin | polyclonal | rabbit | IF | No | 1:50 |
| CD3 | monoclonal | mouse | IF | No | 1:100 |
| CD4 | monoclonal | mouse | IF | No | 1:100 |

CXC: C-X-C motif ligand, FITC: fluorescein isothiocyanate, FCM: flow cytometry, FDC: follicular dendritic cell, ICAM-1: intercellular adhesion molecule-1, IF: immunofluorescence, VCAM-1: vascular cell adhesion molecule-1.
