# Supplementary material for: Inhibitory effect of HTLV‐1 infection on the production of B‐cell activating factors in established follicular dendritic cell‐like cells
Source: Immun Inflamm Dis. 2021 May 4;9(3):777–91. doi: 10.1002/iid3.432 (PMC8342235; doi:10.1002/iid3.432)
Supplement: Supplementary file 6 — Supporting information. [file IID3-9-777-s001.docx]

**Suppl. Fig. S1. The expressions of CXCR5 and Gag protein on HTLV-1-infected cell lines. A:** The CXCR5 expressions on HCT-5 cells, MT-2 cells, and MOLT-4 cells revealed by FCM. CXCR5 was stained with mouse anti-CXCR5 monoclonal antibody followed by FITC-conjugated anti-mouse secondary antibody. **B,C:** The Gag expressions on each cell line were examined by FCM **(B)** and IF **(C)**. Gag was stained with mouse anti-Gag monoclonal antibody followed by FITC-conjugated anti-mouse secondary antibody. Bar: 10▒μm.

**Suppl. Fig. S2. The expressions of BAFF and CXCL13 on FDC-like cells at 24- and 72-hr direct co-culture.** The expressions of BAFF and CXCL13 on unstimulated and stimulated FDC-like cells at 24 and 72▒hr shown by immunofluorescence. **A:** The expression of BAFF at 24▒hr and 72▒hr of direct co-culture with HCT-5 cells in the presence of 1 μg/ml of IFNα/γ or 10▒ng/ml of TNF-α /LTα1β2. BAFF was stained with mouse anti-BAFF monoclonal antibody followed by FITC-conjugated anti-mouse secondary antibody. The expression of BAFF on HCT-5 aggregates is also shown. Mouse IgG (mIgG): internal control. **B:** The expression of CXCL13 at 24▒hr and 72▒hr of direct co-culture with HCT-5 cells in the presence of 1 μg/ml of IFNα/γ or 10▒ng/ml of TNF-α /LTα1β2. CXCL13 was stained with goat anti-CXCL13 polyclonal antibody followed by FITC-conjugated anti-goat secondary antibody. The expression of CXCL13 on HCT-5 aggregates is also shown. Normal goat serum (NGS): Internal control. Representative results of four independent experiments with similar results are shown. **C:** The expressions of BAFF and CXCL13 on six aggregates of HCT-5 cells as merged views. FITC-conjugated anti-mouse secondary antibody and TRITC-conjugated anti-goat secondary antibody were used as secondary antibodies. Bar: 10▒μm. LT: lymphotoxin.

**Suppl. Fig. S3. The MFI of BAFF and CXCL13 on FDC-like cells by immunofluorescence at 24- and 72-hr direct co-culture.** The expressions of BAFF and CXCL13 on unstimulated and stimulated FDC-like cells at 24 and 72▒hr are shown. **A:** The expression of BAFF at 24▒hr and 72▒hr of direct co-culture with HCT-5 cells in the presence of 1 μg/ml of IFNα/γ or 10▒ng/ml of TNF-α /LTα1β2. **B:** The expression of CXCL13 at 24▒hr and 72▒hr of direct co-culture with HCT-5 cells in the presence of 1 μg/ml of IFNα/γ or 10▒ng/ml of TNF-α /LTα1β2. Representative results of four independent experiments with similar results are shown. Data are mean (SD). MFI: mean fluorescence intensity.

**Suppl. Fig. S4. The expressions of BAFF and CXCL13 in indirect culture supernatant. A:** The expression of BAFF in supernatant from FDC-like cells in the absence or presence of various concentrations of HCT-5 cells was examined by ELISA. **B:** The expression of CXCL13 in supernatant from FDC-like cells in the absence/presence of various concentrations of HCT-5 cells was examined by ELISA. In panels A and B, the indirect co-culture of FDC-like cells and HCT-5 cells was performed by using a 0.4-μm-pore Transwell. The samples of co-cultured medium were centrifuged at 10,000▒rpm for 5▒min at 4℃, and then the supernatant was collected in new tubes. FDC culture medium and HCT-5 culture medium were also measured as internal controls. **C:** Morphological changes of an FDC-like cell under the presence of 1 μg/ml of IFN-α, IFN-γ, or both for 48▒hr. The morphology with IFN-α and IFN-γ under the presence of 1×10^6^ HCT-5 cells/ml is also shown. **D:** Bright-field views of FDC-like cells after direct (*lower panels*) or indirect (*upper panels*) co-culture with various HCT-5 cell counts. Indirect co-culture with HCT-5 cells was performed through a Transwell permeable support. FDC-like cells were also directly or indirectly cultured in HCT-5 culture medium (original magn. 200×). Representative results of four independent experiments with similar results are shown. n.d.: not detected.
